# Recurrent Hypophosphatemia Following a Single Dose of Parenteral Iron Administration

**DOI:** 10.7759/cureus.73967

**Published:** 2024-11-19

**Authors:** Swaroopa Kinnera, Oluwaseyi Owasoyo, Hafiz Muhammad Zahid Rahim, Asma Rahim, Kashish Keswani Sunil, Prabhav Chaudhary, Uzma Rahim, Abrar A Awan

**Affiliations:** 1 Acute Medicine, Royal Stoke University Hospital, Stoke-on-Trent, GBR

**Keywords:** calcitriol, ferric carboxymaltose, fibroblast growth factor 23, iron deficiency anaemia, iron induced hypophosphataemia, mechanism of phosphate regulation, phosphatonins

## Abstract

Although parenteral iron is widely used to treat iron deficiency anemia (IDA), some side effects have been inadequately explored. Hypophosphatemia is becoming a well-documented, yet poorly understood, side effect of parenteral iron infusion, oftentimes causing serious and/or prolonged complications.

In this article, we discuss the case of a 33-year-old female with IDA who suffered debilitating physical and mental symptoms of significant recurrent hypophosphatemia following a single standard dose of parenteral iron administration. Despite initial management with repeated parenteral and oral phosphate replacement, the hypophosphatemia, along with its symptoms and sequelae, persisted until active vitamin D supplementation with calcitriol was commenced following a multispecialty team decision.

This case highlights the deficiencies in the recognition and management of parenteral iron-induced hypophosphatemia and proposes a requirement for standardization in its management strategies, including the use of vitamin D supplementation.

## Introduction

Iron deficiency anemia (IDA) is a leading cause of anemia, affecting approximately more than two billion people worldwide [1]. In everyday practice, it is often treated with oral iron preparations. However, unsuccessful oral therapies or severe cases of IDA are commonly treated with parenteral iron, including ferric derisomaltose (FDI), which is also known as iron isomaltoside; ferric carboxymaltose (FCM); and ferumoxytol (FER), which are the newest forms of IV iron preparations [2].

According to the British National Formulary (BNF), hypophosphatemia is a common or very common side effect of parenteral iron infusion [3]. However, the mechanism of action, duration, intensity, and management of drug- or iron-induced hypophosphatemia (IIH) are still poorly understood.

Phosphate acts as a vital element in many biological mechanisms, including cellular membrane integrity, bone mineralization, and signal transduction [4]. Parathyroid hormone and vitamin D play crucial roles in phosphate balance; however, phosphate metabolism and regulation are better recognized with advancements in modern medicine [4].

This article discusses the detailed pathophysiology of phosphate metabolism and the interactions between parenteral iron supplements and phosphate metabolism.

## Case presentation

A 33-year-old female of White British ethnicity with a WHO performance status of 1 presented with a sudden onset history of her legs giving way associated with generalized weakness, loss of appetite, and worsening mobility. She also mentioned a single episode of sudden onset atypical non-cardiac chest pain, with no association of any exertional, infective, and FAST (face, arms, speech, and time)/stroke-like symptoms.

Her past medical history included IDA, Lyme disease, attention deficit hyperactivity disorder, obsessive and compulsive disorder, silent migraines, and posterior fossa arachnoid cyst. She was on regular oral ferrous sulfate 200 mg once daily (OD) and amitriptyline 10 mg once at night (ON). Furthermore, her lifestyle history indicated that she was a non-smoker with no history of alcohol or drug abuse, and no significant risk factors were identified for carcinogenesis.

The general physical examination and focused systemic examinations were always unremarkable. Magnetic resonance imaging (MRI) of the head was reassuring, showing a stable and unchanged posterior fossa arachnoid cyst, a normal spinal cord, and a mild disc bulge at C6/7. The neurology team suspected the symptoms to be psychosocial. After radiological, neurological, and general pathological assessments, with reassuring results for thyroid functions, cortisol levels, bone profile (calcium, albumin, phosphate, and alkaline phosphatase), vitamin D, and other baseline investigations, she was discharged with safety netting advice.

A few months later, she presented with a few days' history of fatigue and muscle weakness, having reportedly received an FCM infusion in a private hospital setting seven days earlier. The patient described feeling unwell after the infusion, with progressive muscle weakness and a sensation of pins and needles in all her limbs. She presented to the hospital with these concerns, and further investigation revealed significant hypophosphatemia of 0.4 mmol/L (reference range: 0.8-1.5 mmol/L).

**Figure 1:**
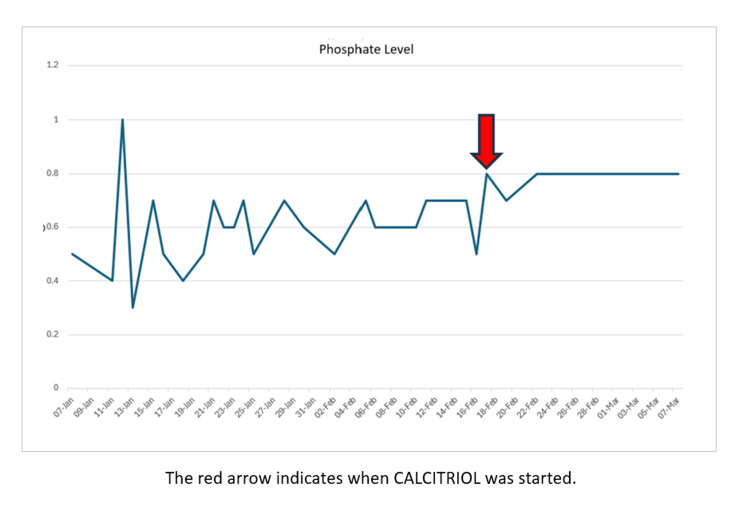
Graph indicating phosphate levels across a span of two months on repeated phosphate and calcitriol supplementation

According to hospital/trust guidelines, she was treated with a parenteral phosphate infusion with 0.2-0.5 mmol phosphate/kg (equivalent to 2-5 mL/kg) of phosphate polyfusor over 12 hours. The follow-up phosphate levels demonstrated worsening hypophosphatemia, for which parenteral phosphate infusion was repeated. She was thereafter discharged following normalization. However, she unfortunately returned to the hospital two days later, with paresthesia in her lips, fingers, and toes. Her repeat phosphate level was 0.3 mmol/L; so, she was re-admitted for further parenteral phosphate correction. She received two more doses of parenteral phosphate before being discharged with oral phosphate supplements and the acute medical virtual ward support to monitor her phosphate levels. Unfortunately, she continued to experience recurrent episodes of symptomatic hypophosphatemia almost daily after parenteral infusions. Her further investigations reported elevated urinary phosphate concentrations. The red arrow in Figure 1 indicates when calcitriol was started.

During the course of her management, she underwent numerous specialty reviews, including acute internal medicine, hematology, endocrinology, dietary, pharmacy, and mental health liaison specialists. The repeated hospital attendances, phlebotomy, and parenteral infusions affected her overall quality of life, resulting in a notable decline in her mental health and her husband's, as well as fear of the unknown. Furthermore, it also led to a lack of confidence in healthcare, indirectly leading to aggravated psychogenic and mental issues.

Subsequently, after more than a month of almost daily parenteral phosphate replacements and further research, a multispecialty decision was made to discontinue intravenous infusions and commence oral calcitriol 0.5 micrograms OD for three days. An outpatient follow-up plan was formulated to monitor daily phosphate levels until stable values were achieved for three consecutive days. Afterward, phosphate levels were monitored every three days for a week, with subsequent dose adjustments of oral calcitriol 0.5 micrograms to once every three days or weekly, depending on phosphate levels. The phosphate levels stabilized one week after starting vitamin D supplementation. Once stable, the patient was followed up with weekly phosphate level checks for another two weeks.

If there were any negative fluctuations, she was managed with short courses of oral phosphate supplements and continued on over-the-counter vitamin D supplements, although the quantity and dosage were never revealed.

## Discussion

Hypophosphatemia is characteristically defined as mild (<2.5 mg/dL/0.8 mmol/L), moderate (<2.5-2 mg/dL/<0.8-0.6 mmol/L), severe (<2-1 mg/dL/<0.6-0.3 mmol/L), and potentially life-threatening (<1 mg/dL/0.3 mmol/L) [5]. Symptoms of hypophosphatemia range from mild to moderate, such as tiredness, bone pain, and muscle weakness, which can mimic symptoms of IDA [2], to more serious symptoms, such as asthenia, myopathy, respiratory failure, and bone complications [5].

Serum and extracellular fluid concentrations of phosphate are regulated by the kidneys and intestines [6]. In the kidneys, parathyroid hormone decreases phosphate reabsorption in the proximal tubules via cyclic adenosine monophosphate (cAMP) dependent mechanisms, while some studies show that 1,25(OH)2D3 has the opposite effect by increasing the reabsorption of phosphate from the proximal and, perhaps, the distal tubule [6]. In the intestines, however, while phosphate absorption depends on dietary ingestion, the efficiency of phosphate absorption is increased by 1,25(OH)2D [6].

Numerous recent studies have described additional mechanisms for phosphate regulation, via cAMP-independent means [4,7]. These include several proteins (fibroblast growth factor 23 (FGF-23), secreted frizzled-related protein 4 (sFRP-4), matrix extracellular phosphoglycoprotein, and FGF 7 (FGF-7)) that decrease renal sodium-dependent phosphate transport [4,7].

These proteins are termed "phosphatonins" and act to reduce the direct reabsorption of renal phosphate in proximal tubules and indirectly inhibit the 1-alpha, 25(OH)2D3 by reducing its synthesis, eventually impeding intestinal absorption of phosphorus [4]. They have thus been implicated in a range of hypophosphatemia-related clinical pathologies/disorders [4].

FGF-23 is synthesized in osteocytes and acts as a regulator of phosphate homeostasis via a complex interaction between the bones, kidneys, parathyroid glands, and the intestines [8,9]. In conjunction with klotho, FGF-23 (usually the intact form iFGF-23) acts by downregulating the expression of NaPi-2a and NaPi-2c, which are the primary Na+-dependent transporters of phosphate in the proximal tubule, thereby increasing renal phosphate losses [8,9]. Additionally, FGF-23 also acts to reduce the conversion of inactive vitamin D to active vitamin D (calcitriol) by acting on enzyme 25-hydroxyvitamin D3 1-alpha (25OHD-1a) hydroxylase via reduced renal cytochrome P450 (Cyp)27b1 expression [8,9]. The resultant effect of reduced active vitamin D leads to a fall in NaPi-2b transporters in the gut, thus reducing the amount of phosphate absorbed [8,9]. Furthermore, this decrease in circulating active vitamin D levels causes slight hypocalcemia, which increases parathyroid hormone levels, further worsening hypophosphatemia owing to its phosphaturic nature [10].

Hypophosphatemia is an increasingly recognized side effect of IV iron treatment, occurring within the first two weeks following administration [5], with ferric carboxymaltose (FCM) being consistently associated with higher rates of hypophosphatemia [2]. Van Doren et al. summarized numerous clinical trials, meta-analyses, and systemic reviews that highlighted FCM causing hypophosphatemia with an incidence range of 47%-75% and a prolonged duration of up to six months and beyond, following administration, compared to <10% incidence with other IV iron preparations: low molecular weight iron dextran (LMWID), ferumoxytol, and FDI [5]. More evidence from two PHOSPHARE-IDA trials identified significantly higher incidences of hypophosphatemia in patients treated with FCM versus FDI, with persistent incidences of hypophosphatemia in 57.3% of patients treated with FCM, which was not observed in FDI-treated patients [2].

It has been suggested in previous studies involving rats and mice that FGF-23 is the primary factor implicated in parenteral IIH [9]. IDA favors stable phosphate levels by stimulating transcription and degradation of FGF-23 to cFGF-23, which is its inactive form. However, for reasons not fully understood, FCM characteristically inhibits the cleavage of FGF-23, leading to an increase in active FGF23 (iFGF23) levels, which promotes phosphaturia [9]. It is also suggested that FCM promotes secondary hepatic and lymphatic iFGF23 production without corresponding cleavage, leading to elevated circulating active FGF23 and further worsening hypophosphatemia [9].

Extensive research into the underlying mechanism of IIH [2] suggests increased urinary phosphate excretion [11] due to a sharp 3-6-fold increase in iFGF23, starting within the first 24 hours after FCM infusion [5,11]. This results in hyperphosphaturic hypophosphatemia, eventually leading to reduced 1,25 (OH)2 vitamin D levels, hypocalcemia, and secondary hyperparathyroidism [2,5], which further increases urinary phosphate excretion [11]. Apart from its phosphaturic effects, FGF23 also directly inhibits the activation of 25 (OH) vitamin D to 1,25 (OH)2 vitamin D [11]. This biochemical cascade triggered by FCM infusion has therefore been termed the “6H-syndrome: (1) high FGF23, (2) hyperphosphaturia, (3) hypophosphatemia, (4) hypovitaminosis D, (5) hypocalcemia, and (6) secondary hyperparathyroidism” [2].

**Figure 2:**
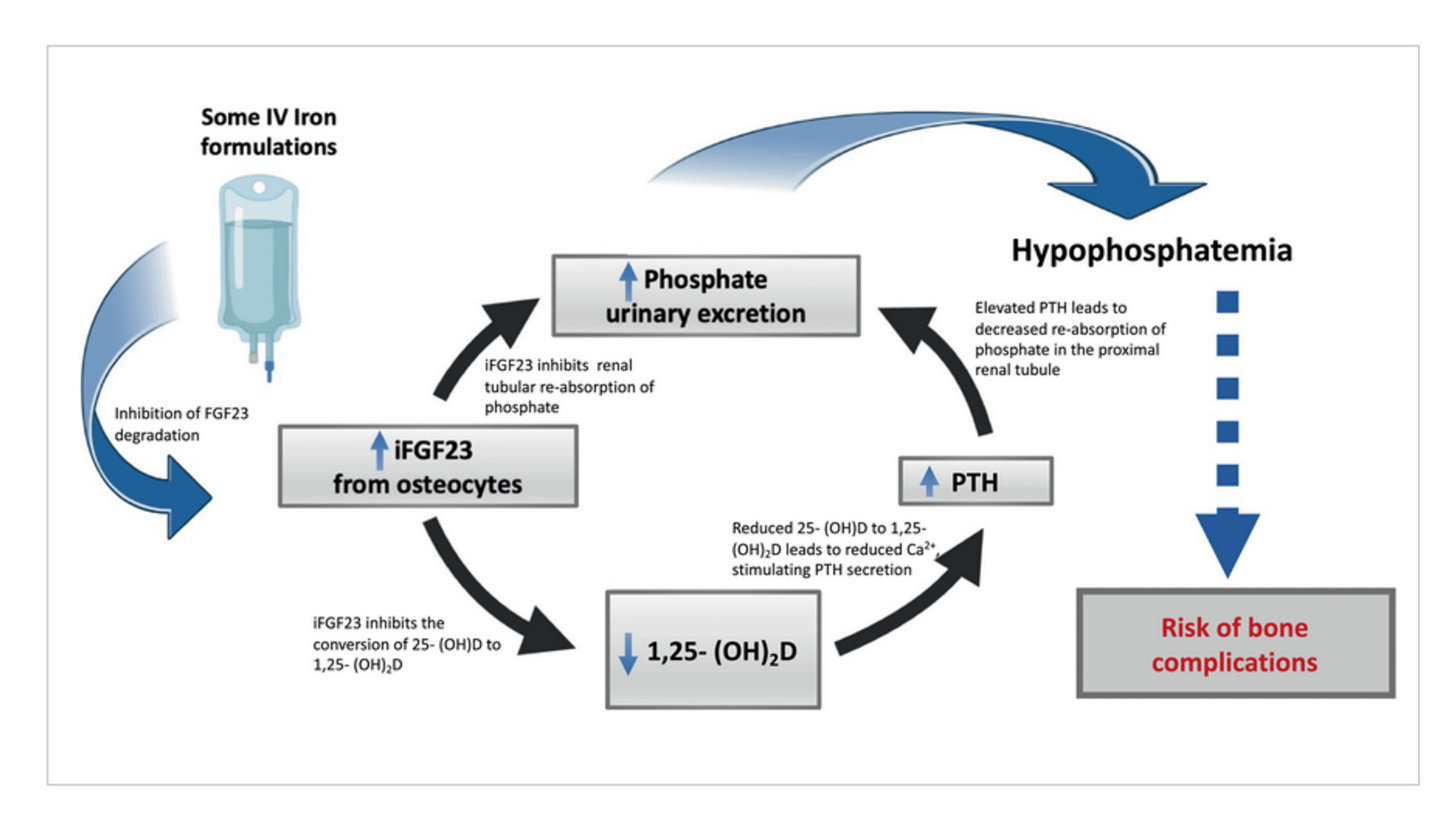
Mechanism of action of iron-induced hypophosphatemia PTH: Parathyroid hormone; FGF: Fibroblast growth factor. Source: Ref. [2].

This sequence of events results in increased bone turnover with prolongation of adverse metabolic bone diseases including osteomalacia, fractures, and other bone deformities [2,5,11]. This is particularly worrisome for patients requiring repeated courses of IV iron, usually on account of recurrent blood loss or maladaptive states [5]. Data from a study describing the symptoms and complications of IV IIH highlighted that patients with gastrointestinal or gynecological blood loss were more often reported with symptomatic hypophosphatemia than patients with underlying kidney diseases [11]. Interestingly, according to the observations, GFR was directly proportional to the severity of hypophosphatemia, making patients with normal kidney functions at a higher risk of developing severe hypophosphatemia and patients with chronic kidney disease with no or mild risks [5]. A considerable risk was identified between phosphate levels being inversely proportional to the severity of IDA [5]. Figure 2 presents the mechanism of action of iron-induced hypophosphatemia.

Perhaps due to lacunae in knowledge, there exists no standard practice or care of management for IIH [5]. There appears to be no consensus on the diagnosis and management of IIH. While some studies recommend pre- and post-infusion testing of phosphate levels in all patients receiving iron infusion [2], others do not recommend universal screening but rather recommend measurement of serum phosphate exclusively in symptomatic patients, as well as pre-testing of serum phosphate levels before additional infusion doses in patients requiring multiple infusions [11].

Detlie et al. and Schaefer et al. established that parenteral IIH is refractory to oral and parenteral phosphate substitutes, and subsequent supplementation may lead to worsening hypophosphatemia [11,12], as phosphate supplementation is likely to increase urinary phosphate losses rather than correct the hypophosphatemia, considering the iFGF23-mediated pathophysiology at play [10]. However, Kassianides and Bhandari suggested a combination of calcitriol in conjunction with oral and parenteral phosphate supplementation to mitigate FGF-23-associated hypophosphatemia [9], as in the case of parenteral IIH.

Therefore, the management of asymptomatic hypophosphatemia should be restricted to observation and the use of active vitamin D substitutes to balance out the physiological effects of secondary hyperparathyroidism [11]. Furthermore, parenteral iron cessation is advised to avoid phosphaturia and subsequent worsening of hypophosphatemia. Recurrent treatment with parenteral iron infusion also mandates serum phosphate monitoring with safety netting signs and symptoms of severe hypophosphatemia in any patient receiving iron infusion within three months. Management should therefore be guided by the severity and presentation, as the risk and duration of hypophosphatemia are impossible to predict [10]. The studies also reported the need for imaging in patients complaining of bone pain following iron infusion [11,12].

Additionally, burosumab, a therapeutic monoclonal antibody against FGF-23, is purported to present a possible treatment option [2]. Although it has not been licensed for this indication, it has been reportedly successfully utilized in a single patient and could be considered useful in severe cases [11]. Also, although calcimimetics such as cinacalcet are effective in managing tumor-induced osteomalacia, they are also not improved for IV IIH [11] but are certainly worth considering. There also appear to be no concrete conclusions as to the role, feasibility, and cost implications of FGF-23 testing in the context of parenteral IIH; therefore, further research into this is required.

## Conclusions

In conclusion, persistent IIH is a well-recognized and common, yet often neglected and underestimated, side effect of parenteral iron infusion, which can cause potentially debilitating side effects. This report is a classic example of overzealous management with gaps in the existing knowledge. It is worth stressing that there is a need to establish standardized guidelines for the workup, follow-up, and management of IIH, especially in patients receiving multiple iron infusions. The best management for existing IIH involves the use of active vitamin D substitutes, with or without concurrent oral or parenteral phosphate supplementation.
